# Targeting inflammasome, IL-1β, and coagulation in melioidosis

**DOI:** 10.3389/fmicb.2026.1816008

**Published:** 2026-04-28

**Authors:** Guilherme Melo, Fabio Re

**Affiliations:** Department of Microbiology and Immunology, Rosalind Franklin University of Medicine and Science, North Chicago, IL, United States

**Keywords:** *Burkholderia pseudomallei*, coagulation, inflammasome, inflammation, melioidosis, neutrophils, pyroptosis, sepsis

## Abstract

*Burkholderia pseudomallei* is a Gram-negative bacterium that causes melioidosis, a disease endemic in Southeast Asia and Northern Australia and increasingly detected in other tropical regions. *B. pseudomallei* is resistant to many antibiotics and no vaccine for melioidosis is currently available. The disease, particularly its pneumonic form, has high mortality, often due to sepsis. Accumulating evidence from our and other groups has shown that inflammasome and toll-like receptors (TLR) activation during *B. pseudomallei* lung infection causes an excessive inflammatory response that becomes deleterious due to damage to lung tissues. Interleukin (IL)-1β and neutrophils proteases appear to be particularly detrimental. Caspase-11 inflammasome activation has been shown to play a critical role in sepsis through activation of Tissue Factor and blood coagulation leading to disseminated intravascular coagulation. Sepsis and coagulopathy are serious complications in melioidosis suggesting that caspase-11 activation can be a pathogenic mechanism in this disease. Here, we discuss the potential therapeutic benefits of inhibitors of inflammasomes, IL-1β, neutrophil elastase, bradykinin and Tissue factor for melioidosis treatment.

## 1 Introduction

Meliodosis is emerging as an interesting disease to investigate the dual role played by inflammation, coagulation, and caspase-11. *Burkholderia pseudomallei*, a Gram-negative, rod-shaped, motile, flagellated bacterium, is the etiological agent of melioidosis, a serious and often fatal tropical infectious disease endemic in Southeast Asia and Northern Australia (Cheng and Currie, 2005; Wiersinga et al., 2018; Gassiep et al., 2020; Meumann et al., 2024) and increasingly detected in other tropical regions. It has been estimated that the global burden of melioidosis can be as high as 165,000 cases per year, suggesting that this disease is severely underreported (Limmathurotsakul et al., 2016). Due to its high mortality rate, pathogenic potential, and resistance to several antimicrobials, *B. pseudomallei* is classified as a Tier 1 Select Agent by the CDC and NIAID. *B. pseudomallei* is commonly found in contaminated water and soil, and human infection is mostly contracted through subcutaneous inoculation, ingestion, or inhalation of aerosolized bacteria. *B. pseudomallei* infects macrophages and other cell types and is able to survive and replicate inside the cytoplasm by deploying an array of virulence factors and type III, type IV, and type VI secretion systems (Lazar Adler et al., 2009). *B. thailandensis* is a related *Burkholderia* species not pathogenic to humans but that causes a lethal disease in mice, and is often used as a mouse model of melioidosis (Brett et al., 1998; Haraga et al., 2008). Other *Burkholderia* species pathogenic to mammals are *B. cepacia* complex, which includes bacteria that infect cystic fibrosis patients, and *B. mallei*, which causes glanders in equids. Although most immunocompetent individuals that contract *B. pseudomallei* clear the bacterial infection and undergo a subclinical disease, those who develop melioidosis often face a severe and lethal spectrum of disease forms. In these patients the infection spreads systemically with pneumonia, organ abscesses, and bacteremia being the most common and severe clinical presentations. Sepsis is a frequent complication in melioidosis and the main cause of death (Chaowagul et al., 1989). In addition, a minority of infected individuals (11%) can exhibit symptomatic chronic melioidosis or show asymptomatic persistence in the host with a reported latency period of up to 29 years before reactivation and disease relapse (Ngauy et al., 2005). Risk factors for melioidosis include diabetes, alcoholism, and chronic pulmonary and renal diseases (Gassiep et al., 2020). Currently no vaccine is available for melioidosis and treatment includes a prolonged and intense regimen of antibiotics. The lack of an effective vaccine for melioidosis and the bacterium resistance to several antibiotics underscore the need to develop host-targeted interventions. It is increasingly recognized that excessive inflammation and dysregulated coagulation are associated with the severity of melioidosis cases and, therefore, appear as plausible therapeutic targets that can improve the infection outcome.

## 2 Innate immunity, inflammation, pyroptosis, and coagulation

Activation of the innate immune response is essential for protection from infections. To detect infections or tissue damage the innate immune system relies on families of pattern recognition receptor (Li and Wu, 2021) like the Toll-like receptors (TLR) and the Nod-like receptors (NLR) that compose the inflammasome (Li and Wu, 2021). These receptors are expressed on cells of the immune system and other tissues and recognize conserved microbial components known as pathogen-associated molecular patterns (PAMPs) and host-derived signals called damage-associated molecular patterns (DAMPs), initiating rapid immune responses and inflammation.

TLR localized at the cell surface or in the endosomal compartment recognize bacterial components, like the LPS, lipoproteins, and nucleic acids, leading to production of a wide variety of cytokines, chemokines, adhesion molecules and other mediators that initiate the inflammatory response (Fitzgerald and Kagan, 2020). The NLR function as cytoplasmic sensors of bacterial components or of signs of infection and assemble into large multi-protein complexes called inflammasomes that control activation of caspase-1 (Broz and Dixit, 2016). This protease cleaves Gasdermin D (GSDMD), a cellular protein that assembles at the cell membrane forming a large pore leading to a lytic form of cell death termed pyroptosis. By killing the infected cells, pyroptosis destroy the replicative niche of intracellular pathogens and, therefore, it is highly protective against certain bacterial and viral infections (Aachoui et al., 2013; Li et al., 2023). GSDMD cleavage and pyroptosis are also triggered by activation of the non-canonical inflammasome composed of caspase-11 in mice or caspase-4 and caspase-5 in humans (Oh et al., 2020), proteases that directly recognize LPS localized in the cytoplasm (Hagar et al., 2013; Kayagaki et al., 2013). Several studies have documented the protective role of Caspase-11-dependent pyroptosis, particularly in myeloid cells but also in lung and intestinal epithelial cells (Hagar and Miao, 2014; Wang et al., 2018; Kovacs et al., 2020; Oh et al., 2022; Casson et al., 2013; Knodler et al., 2014; Crowley et al., 2020; Aachoui et al., 2015). Activation of caspase-1, but not caspase-11, also cleaves the immature form of pro- interleukin (IL)-1β and pro-IL-18, converting them into the secreted bioactive pro-inflammatory cytokines. By rapidly recruiting PMN and other leukocytes to the site of infection and activating various innate immune mechanisms, inflammation is an essential protective response, particularly in the early phase of the infection but also at later time points instructing dendritic cells maturation and lymphocytes activation and differentiation.

Blood coagulation is another defensive mechanism activated by infection. Its physiologic purpose is not only haemostasis following injury but also to trap bacteria and other pathogens in the fibrin clot (Nickel et al., 2025) preventing systemic dissemination and allowing more efficient killing and clearance of microbes in a process termed immunothrombosis (Engelmann and Massberg, 2013). In addition, coagulation and inflammation reciprocally activate each other and, therefore, the coagulation cascade is increasingly recognized as a component of the innate immune system (Esmon, 2005). Inflammatory cells like monocytes/macrophages and neutrophils participate in the activation of coagulation and platelet leading to formation of small intravascular thrombi with innate immune functions. However, immunothrombosis and inflammation may become pathogenic when excessive and dysregulated.

The coagulation contact system (Long et al., 2016; Renne and Stavrou, 2019) can be activated by direct interaction of coagulation factor XII (FXII) with bacterial-derived polyphosphates or by binding to the surfaces of neutrophils or to neutrophil extracellular traps (NETs) (Renne and Stavrou, 2019; Muller et al., 2009). This leads to the initiation of the coagulation cascade and fibrin deposition but also to the release of bradykinin, a potent proinflammatory peptide derived from cleavage of high molecular weight kininogen by plasma kallikrein (Schmaier, 2016). Release of bradykinin causes increased vascular permeability, vasodilation, hypotension, pain, and fever. Bradykinin is also chemotactic for neutrophils and triggers their degranulation (Wachtfogel et al., 1986). The contact system is viewed as an amplification mechanism that contributes to pathogenic immunothrombosis but not to hemostasis (Long et al., 2016), which is mainly initiated by activation of the extrinsic pathway of coagulation (Mackman et al., 2007). This pathway depends on tissue factor (TF, factor III), a protein that is present on the sub-endothelial space as well as on circulating monocytes and neutrophils. Upon vascular injury, TF comes in contact with blood and, in a process called decryption (Butenas and Krudysz-Amblo, 2012), it is activated and released in soluble form or embedded into microvesicles. Binding of activated TF to factor VII then initiates the coagulation cascade that culminates in the thrombin-mediated conversion of fibrinogen into fibrin and formation of a solid clot. TF is strongly induced by inflammatory stimuli in myeloid and endothelial cells (Sachetto and Mackman, 2023). To avoid harmful outcomes due to deregulated activation, the coagulation cascade must be finely regulated at many levels by a number of proteins including the TF pathway inhibitor (TFPI), the antithrombin (AT), the thrombomodulin (TM)/Activated Protein C (APC), and by the plasminogen/plasmin fibrinolytic system.

## 3 Inflammation, pyroptosis, and coagulation detrimental effects

While inflammation, pyroptosis, and coagulation are potentially protective responses against infections, they may also become damaging to the host tissues and, if not properly regulated, cause to serious chronic pathologies and even life-threatening conditions. The lung appears to be particularly vulnerable to aberrant activation of inflammation and coagulation as best exemplified during sepsis.

### 3.1 Sepsis and caspase-11

Sepsis is defined as a life-threatening organ dysfunction caused by an unregulated host response to infection (Singer et al., 2016; Angus and van der Poll, 2013). Sepsis is the main cause of death in hospitalized patients worldwide responsible for 11 millions death every year. This disease is characterized by an early phase of overwhelming inflammation caused by infection or severe trauma followed by an immunosuppression phase. The initial cytokine storm caused by massive PRR activation leads to increased vascular permeability and endothelial cell barrier break-down resulting in shock. This is coupled to TF-driven systemic thrombin generation and to suppression of fibrinolysis and TFPI activity promoting a procoagulant state that leads to clogging of small vessel in a process called disseminated intravascular coagulation (DIC) (Levi and van der Poll, 2017). The resulting ischemia causes multi organ failure and death. The later phase of sepsis resembles an immunoparalysis that increases susceptibility to secondary infection and impairs the host's ability to clear the infection. Consumption of platelets and coagulation factors also results in a sustained susceptibility to bleeding and predispose to relapses.

While several aspects of sepsis and the immunothrombosis resulting from the crosstalk between innate immunity, inflammation, and coagulation remain unclear, recent papers have brought some clarity and identified caspase-11 as a key player in these processes (Tang et al., 2021; Ryan et al., 2022). Caspase-11 deficient mice were shown to be resistant to LPS-induced endotoxic shock (Hagar et al., 2013; Kayagaki et al., 2013). It was later shown that caspase-11-mediated pyroptosis of endothelial cells exacerbated neutrophils recruitment and destruction of lung endothelial barrier in a model of endotoxin acute lung injury (Cheng et al., 2017). Finally, it was shown that caspase-11 activation and GSDMD pore formation resulted in TF decryption and activation of the coagulation cascade in a cell death-independent fashion and pharmacologic inhibition of caspase-11 activation reduced coagulopathy, organ injury, and mortality (Wu et al., 2019; Yang et al., 2019; Peng et al., 2020). In these studies, levels of IL-1β were shown to correlate with DIC in sepsis patients supporting a potential link between deregulated inflammasome activation and coagulation in melioidosis.

However, it should be noted that the caspase-11-dependent activation of TF and DIC has been observed only in the experimental model of LPS endotoxemia. Although it is likely that this pathway may be activated during infection, it is still unclear in which circumstances this may occur and how it may impact the overall response to the infection. Future work in this area remains a priority.

## 4 Excessive inflammation is deleterious in melioidosis

Infection with *B. pseudomallei* is associated with a strong inflammatory response and in melioidosis patients elevated plasma levels of cytokines, including several associated with neutrophils recruitment like IL-1β, IL-6, TNFα, CXCL8, IL-17a, correlate with poor prognosis (Simpson et al., 2000; Kaewarpai et al., 2020; Wright et al., 2021; Khemla et al., 2025). Other indicators of high inflammatory response, like HMGB1, soluble TRIM-1 and MIF were also elevated in melioidosis patients and positively correlated with disease severity and mortality (Wiersinga et al., 2007a, 2010; Charoensup et al., 2014). Sepsis is one of the most common and severe complication of melioidosis and interventions to mitigate its occurrence should be a priority.

### 4.1 TLR and inflammasome activation in melioidosis

TLR and inflammasome are critically participating to the innate immune response to *B. pseudomallei* infection leading to production of several proinflammatory cytokines. While *B. pseudomallei* LPS has been reported to be a relatively weak TLR4 agonist compared to other Gram-negative bacteria LPS (Novem et al., 2009; Weehuizen et al., 2015), other studies found that it is still able to potently activate TLR4 (Chantratita et al., 2013; Sengyee et al., 2018, 2019). TLR2 and TLR5 are also strongly activated by *B. pseudomallei* (West et al., 2014; Birnie et al., 2019; Amemiya et al., 2021). The mouse model of pneumonic melioidosis has been very useful to support the notion that excessive inflammation is detrimental to the host during melioidosis. Mice deficient in MyD88, a signaling component for TLR and IL-1β and IL-18 receptors, are more susceptible to *B. pseudomallei* infection (Wiersinga et al., 2008c), suggesting a protective role of inflammation in melioidosis. In contrast, TLR2 deficient mice showed increased resistance while absence of TLR4 did not affect the outcome of the infection (Wiersinga et al., 2007b). Absence of CD14 was also protective (Wiersinga et al., 2008a). Moreover, a hypofunctional TLR5 genetic variant was found to be associated with reduced organ failure and improved survival in melioidosis patients (West et al., 2013; Chaichana et al., 2017) though another study failed to confirm this association (Yimthin et al., 2024). These results suggest that inflammation plays a dual role in melioidosis with a physiologic MyD88-dependent response being protective but often becoming excessive and detrimental, precipitating sepsis.

Inflammasome activation has been shown in human macrophages infected with *B. pseudomallei* (Lichtenegger et al., 2020). In mice infected with *B. pseudomallei or B. thailandensis* inflammasome activation and release of IL-1β were shown to play a dual role (Sahoo et al., 2016) (see Table 1). Mice deficient in the NLR molecules NLRP3 or NLRC4 were dramatically more susceptible to *B. pseudomallei* infection than wild type mice showing decreased survival and increased bacteria burden in lung, spleen, and liver (Ceballos-Olvera et al., 2011). Levels of a number of proinflammatory cytokines and chemokines in BALF and serum correlated with the bacteria burden and were significantly elevated in both knockout mouse strains compared to wild type controls. However, the level of IL-1β and IL-18 were drastically decreased in *Nlrp3*^−/−^, but not *Nlrc4*^−/−^, mice that produced these cytokines at high level. Conversely, pyroptosis was similarly induced in wild type and NLRP3-deficient macrophages but not in NLRC4-deficient cells infected *in vitro*. Thus, the heightened susceptibility of *Nlrp3*^−/−^ mice appeared to be due to decreased production of IL-18 and IL-1β while that of *Nlrc4*^−/−^ mice may be due to decreased pyroptosis and inability to restrict bacteria replication. Both Caspase-1 (Breitbach et al., 2009) and Caspase-11 (Aachoui et al., 2013; Wang et al., 2018, 2019; Srisaowakarn et al., 2020) have also been shown to have a protective role in melioidosis, again indicating the effectiveness of pyroptosis to restrict *B. pseudomallei* replication. Of note, pyroptosis in *B. pseudomallei* or *B. thailandensis* infected macrophages was shown to be primarily mediated by caspase-1 (Wang et al., 2018) while caspase-11 appears to be critical for pyroptosis of infected neutrophils (Kovacs et al., 2020) and lung epithelial cells (Wang et al., 2018).

**Table 1:** Activation of inflammasomes by *Burkholderia* species pathogenic to mammals.

*Burkholderia* sp.	Virulence factor	Inflammasome	Effect on host response	Host	References
*B. pseudomallei*	Phagosome escape Membrane damage	NLRP3	IL-1β release deleterious IL-18 protective	Mouse *in vivo*	Ceballos-Olvera et al., 2011
*B. pseudomallei*	BsaK, T3SS	NLRC4	Pyroptosis, and IL-18, protective	Mouse *in vivo* Mouse BMM	Miao et al., 2010; Ceballos-Olvera et al., 2011; West et al., 2013
*B. pseudomallei*	BsaL, T3SS	Caspase-1, GSDMD	Pyroptosis and IL-1β release	Primary human macrophages	Lichtenegger et al., 2020
*B. pseudomallei*	LPS	Caspase-11/Caspase-4	Pyroptosis, protection against infection	Mouse Human alveolar epithelial cells	Aachoui et al., 2013; Srisaowakarn et al., 2020
*B. thailandensis*	Phagosome escape Membrane damage	NLRP3	Protective response	Mouse (BMM), Mouse *in vivo*	Aachoui et al., 2015
*B. thailandensis*	BsaK, T3SS	NLRC4	IL-18 release, protective response	Mouse (BMM), Mouse *in vivo*	Zhao et al., 2011; Aachoui et al., 2015
*B. thailandensis*	LPS	Caspase-11	Pyroptosis (myeloid and lung epithelial cells), protection against infection	Mouse (BMM), Mouse *in vivo*	Aachoui et al., 2013; Hagar et al., 2013; Wang et al., 2018; Kovacs et al., 2020
*B. thailandensis*	T3SS, LPS	GSDMD	Pyroptosis, protective	Mouse (BMM), Mouse *in vivo*	Wang et al., 2019
*B. cepacia*	TecA, T6SS	Pyrin	Pyroptosis, protective	Mouse (BMM), Mouse *in vivo*	Aubert et al., 2016

Further analysis of mice deficient in IL-18 or IL-1 receptor yielded some surprising results. As expected, and in agreement with the protective role of MyD88, *Il18*^−/−^ mice were more susceptible to melioidosis (Wiersinga et al., 2007c; Ceballos-Olvera et al., 2011) due to the lower level of IFNγ, a cytokine induced by IL-18 and that was necessary for optimal ROS generation. In contrast, *Il1r1*^−/−^ mice were more resistant to lung infection showing lower bacterial burden, decreased neutrophilic lung inflammation, and increased survival (Ceballos-Olvera et al., 2011). Administration of Anakinra, the IL-1 receptor antagonist (IL-1RA), significantly reduced bacteria burden, neutrophilic inflammation, and protected the mice (Ceballos-Olvera et al., 2011). Confirming the detrimental role of IL-1β and neutrophils, administration of an anti- IL-1β antibody protected *B. pseudomallei* infected mice (Weehuizen et al., 2016).

### 4.2 Detrimental role of neutrophils in melioidosis

Recruitment of neutrophils through activation of the endothelium and induction of CXCR1/CXCR2-binding chemokines are among the earliest events triggered by IL-1β (Arend et al., 2008; Garlanda et al., 2025). Neutrophils are essential for primary containment of bacteria and other pathogens (Liu et al., 2017; Haynes et al., 2024). However, in many conditions, exacerbated activation and recruitment of these cells, particularly in the lung, is associated with disease severity (Abraham, 2003; Zemans et al., 2009; Yang et al., 2025) and neutrophilia is clinical marker commonly associated with poor prognosis in lung infections and sepsis (Haynes et al., 2024). Neutrophils are the predominant cell-type associated with *B. pseudomallei* infection (Laws et al., 2011). Neutrophils isolated from diabetic patients and stimulated *in vitro* with *B. pseudomallei* showed reduced release of antibacterial NETs, which could account for the increased susceptibility to melioidosis of these patients (Riyapa et al., 2012). Although NETs can kill *B. pseudomallei in vitro*, NETs release was not protective in mouse melioidosis infection model (de Jong et al., 2014). In fact, it is unclear how effective neutrophils can be against *B. pseudomallei*. While neutrophils depletion increased mice susceptibility to melioidosis (Easton et al., 2007), *B. pseudomallei* was shown to be intrinsically resistant to killing by neutrophils (Chanchamroen et al., 2009). Activation of NADPH oxidase was reported to be unable to completely restrict *B. pseudomallei* intracellular replication (Wikraiphat et al., 2014). Interestingly, neutrophils are resistant to NLRC4-mediated pyroptosis, though this cell death pathway can be induced through caspase-11 during *B. thailandensis* infection (Kovacs et al., 2020; Oh et al., 2022).

The most effective neutrophil's microbicidal mechanisms, namely ROS and proteases, are also the most damaging to the host tissues. Elastase is the main neutrophil protease and it is strongly microbicidal against bacteria, though it has been shown to be harmful in certain infections (Belaaouaj et al., 1998, 2000). Melioidosis patients have higher levels of nucleosome and neutrophil elastase commonly associated to NETs (de Jong et al., 2014). In DIC patients neutrophil elastase was shown to cause release of an inactive form of TM from endothelial cell membrane impairing the activation of the coagulation inhibitor APC (Hayakawa et al., 2011; Ishii and Majerus, 1985; Ishii et al., 1991). Elastase also cleaves and inactivates TFPI (Massberg et al., 2010). These observations are consistent with the notion that neutrophil can be potentially protective against *B. pseudomallei* but their excessive activation and recruitment to the lung can become deleterious. This was demonstrated by the observation that the decreased neutrophils recruitment to lung and elastase release was an important factor determining the increased resistance of *Il-1r1*^−/−^ mice. Elastase-deficient mice were protected from *B. pseudomallei* infection and administration of sivelestat, an elastase inhibitor, reduced lung tissue damage and protected wild type mice from melioidosis (Sahoo et al., 2014). Supporting the notion that excessive neutrophil recruitment and release of elastase could be detrimental in melioidosis while physiologic neutrophil levels could be protective, adoptive transfer of wild type neutrophils in *Cxcr2*^−/−^ mice exacerbated the disease while transfer of elastase-deficient neutrophils proved protective (Sahoo et al., 2014). In line with these results, depletion of the HMGB1, found in high levels during *B. pseudomallei* infection, was found to be beneficial by reducing the neutrophil-recruiting chemokine CXCL1 and decreasing the bacterial burden (Laws et al., 2015). Recent findings also showed that γδ T cells have a potential protective role in melioidosis by controlling excessive neutrophil recruitment and function (Wright et al., 2024). Excessive release of neutrophil elastase in *B. pseudomallei* infected mice also contributed to the release of the potent pro-inflammatory peptide bradykinin, leading to increased vascular permeability and damage to the endothelial barrier. Consequently, bradykinin inhibition using a receptor inhibitor proved beneficial (Sahoo et al., 2014).

### 4.3 Detrimental role of inflammasome, IL-1β, and neutrophils in other bacterial infections

Across murine models of bacterial pneumonia, excessive inflammasome activation and pyroptosis have been shown to drive neutrophil-mediated lung immunopathology rather than host protection (Haynes et al., 2024). In *Pseudomonas aeruginosa* infections, where IL-1R and NLRC4 signaling has been shown to provide protection (Reiniger et al., 2007; Franchi et al., 2007; Sutterwala et al., 2007; Tolle et al., 2015), unregulated NLRC4 inflammasome activation in alveolar macrophages promoted IL-1β-dependent excessive neutrophil recruitment and lung injury hindering bacterial clearance and increasing mortality (Cohen and Prince, 2013). Genetic or pharmacological inhibition of NLRC4, caspase-1, IL-1β, or IL-18 resulted in improved outcomes (Cohen and Prince, 2013; Faure et al., 2014; Ryu et al., 2017; Rimessi et al., 2015). In *Staphylococcus aureus* necrotizing pneumonia, NLRC4 deficiency also improved bacterial clearance and survival rates (Paudel et al., 2019) while NLRP3 has been shown to induce lung injury (Kebaier et al., 2012). In *Streptococcus pneumoniae* infection, early neutrophil influx is essential for effective bacterial clearance but sustained neutrophil persistence in the lung and NETs formation correlated with disease severity, and promoted increased pulmonary bacterial burden, bacteremia, and mortality (Hahn et al., 2011; Kadioglu et al., 2011; Bou Ghanem et al., 2015; Bhowmick et al., 2013; Moorthy et al., 2016; Sanders et al., 2022). In this model, the absence of NLRP3 or ASC resulted in lower inflammatory cytokine and chemokine production, reduced tissue damage and bacterial burden, and better survival (van Lieshout et al., 2018). Excessive NETs formation was also detrimental in experimental *P. aeruginosa* lung infections (Sung et al., 2022). Deficiency in the antimicrobial peptide CRAMP resulted in excessive neutrophil recruitment, exacerbated lung injury, and impaired host protection during *Klebsiella pneumoniae* (Kovach et al., 2012). Neutrophil elastase was shown to play a deleterious role during infection with *S. pneumoniae*, where it was associated with increased lung tissue damage to alveolar epithelial cells (van der Windt et al., 2012; Domon et al., 2016). In caspase-1 deficient mice, caspase-11 dependent pyroptosis was found to be deleterious to the host during infection with *Salmonella typhimurium* (Broz et al., 2012). Recent work further uncovered a complex crosstalk between the hematopoietic and non-hematopoietic compartments: in *Escherichia coli* pneumonia, caspase-1–dependent epithelial pyroptosis triggered dsRNA-mediated neutrophil necroptosis, which correlated with lung injury, whereas caspase-1 deficiency reduced tissue damage, improved bacterial clearance, and enhanced survival (Luo et al., 2025). Overall, these findings underscore the potential harm of excessive inflammation and neutrophil recruitment to the lungs.

### 4.4 Detrimental role of coagulation in melioidosis and other bacterial infections

Melioidosis often leads to sepsis and melioidosis patients show activation of coagulation pathways with fibrin deposition in lungs, evidence of DIC, and concomitant downregulation of anticoagulant mechanisms. This shift to a procoagulant state is a marker of poor outcome and correlates with mortality (LaRosa et al., 2005; Wiersinga et al., 2008b; Koh et al., 2011b). Several studies have investigated the role of dysregulated coagulation using a mouse model of melioidosis and unveiled a complex scenario (Kager et al., 2014a). Mice deficient in TM, which are unable to generate APC, a major component of the anticoagulant mechanism, were shown to be more susceptible to melioidosis with increased pulmonary inflammation and activated coagulation (Weiler-Guettler et al., 1998; Kager et al., 2014b). Surprisingly, mice overexpressing APC were also found to be more susceptible to melioidosis (Kager et al., 2013b) suggesting that some level of coagulation can be protective, possibly by trapping bacteria in fibrin clot and preventing systemic spread, yet excessive coagulation may lead to sepsis and DIC. This notion was confirmed by the observation that tissue Plasminogen activator (tPA)-deficient mice, which have impaired fibrinolysis, were protected from melioidosis (Kager et al., 2012) while absence of Plasminogen activator inhibitor-1 (PAI-1), which results in lower levels of fibrin deposition, showed increased susceptibility (Kager et al., 2011). A similar phenotype was reported for mice deficient in the antifibrinolytic factor α2-antiplasmin (A2AP) (Kager et al., 2013a). Supporting a detrimental role of coagulation during certain bacterial infections, administration of recombinant APC protected mice from pneumococcal pneumonia (Schouten et al., 2010, 2011).

While no study has yet investigated the role of TF or the contact system in melioidosis, the observations that activation of caspase-11 and GSDMD triggers release and activation of TF leading to sepsis (Yang et al., 2019; Peng et al., 2020; Shi et al., 2022) and that sepsis is a major pathogenic mechanism in melioidosis (Cheng and Currie, 2005; Chaowagul et al., 1989) suggest that caspase-11-dependent TF activation and DIC may be critically involved in the pathogenesis of melioidosis. Ongoing studies in our lab are exploring this possibility.

The role of the extrinsic and the contact pathways has been investigated in several bacterial infection models showing both protective and detrimental effects. Mice expressing very low level of TF or deficient in fibrinolysis inhibitor were more susceptible to infection with *Yersinia enterocolitica*, suggesting that the extrinsic coagulation pathway may be protective during this bacterial infection (Luo et al., 2011). It should be noted that in this model mice were infected via the intravenous or intraperitoneal route with bacteria spreading to spleen and liver but not to lung. In contrast, these “low-TF” mice or mice with hematopoietic-restricted TF deficiency showed decreased coagulation and inflammation and improved survival in the endotoxemia model (Pawlinski et al., 2004). Severe TF deficiency also conferred protection against LPS lethality in the otherwise vulnerable low–protein C mice (Castellino et al., 2011). In *E. coli* peritonitis, selectively inhibiting the TF/FVIIa complex with recombinant nematode anticoagulant protein c2 (rNAPc2) reduced coagulation activation but did not enhance bacterial control, inflammation, or survival (Weijer et al., 2004).

Research into the role of the contact system in bacterial infections has also yielded varied results.

In a model of *S. pneumoniae* pneumonia and *S. aureus* skin infection FXII-deficient mice were unable to control bacteria dissemination and had higher mortality (Nickel et al., 2025). In contrast, FXII deficiency was beneficial in *K. pneumoniae* pneumonia model (Stroo et al., 2017). The role of FXII in protecting against bacterial infections is further emphasized by the discovery that *Acinetobacter baumannii* secretes a protease that inactivates FXII (Waack et al., 2018). By disabling this important immune component, the bacteria can evade being trapped within fibrin clots, ultimately enhancing their ability to thrive and survive in the host environment.

A major challenge in interpreting and translating findings from the endotoxemia models is that the innate immune function of coagulation, which helps trapping bacteria in the fibrin clot, may not provide any advantage in this sterile model, where only the harmful DIC is present. In contrast, in the context of certain infections the protective innate immune functions may prevail over the deleterious coagulopathy. It is also important to note that the route of infection, and therefore the organs mainly targeted, may determine how impactful deregulated coagulation becomes for the host. Thus, the intraperitoneal or intravenous route may yield quite different results from infection of the lung, an organ that appears to be highly vulnerable to coagulopathy. A further complication is represented by the reciprocal regulation of inflammation and coagulation.

In summary, studies in humans and mice have revealed the duality of inflammation, caspase-11 inflammasome activation, and coagulation in sepsis, melioidosis, and other infections. These findings suggest that targeting the excessive activation of these pathways may be beneficial in the treatment of melioidosis.

## 5 Host-targeted interventions for melioidosis

**Figure 1:**
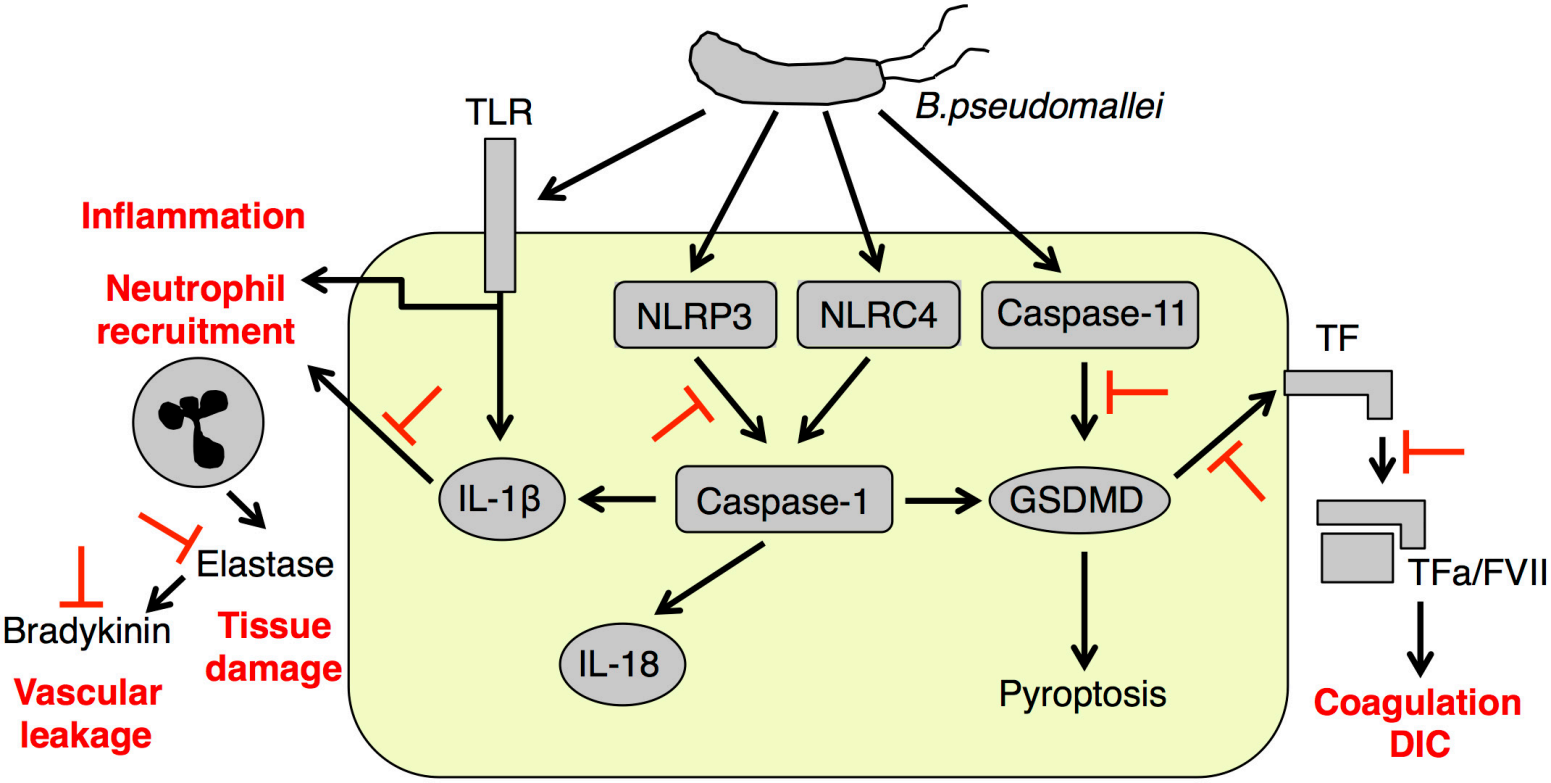
Deleterious pathways in melioidosis as potential therapeutic targets. IL-1β causes excessive neutrophil recruitment to the lung resulting in elastase-mediated tissue damage and bradykinin production. NLRP3 and caspase-11 inflammasomes activate TF leading to coagulation and DIC. Therapeutic targets for melioidosis treatments include NLRP3 and caspase-11, IL-1β, neutrophil elastase, bradykinin, and TF. DIC, disseminated intravascular coagulation; FVII, factor 7; GSDMD, gasdermin D; IL-1β, interleukin-1β; IL-18, Interleukin-18; NLRC4, Nod like receptor C4; NLRP3, Nod like receptor P3; TF, tissue factor; TLR, Toll-like receptor.

Given the critical role played by inflammation, pyroptosis, and coagulation in protecting against bacterial infections, the unrestricted and generalized inhibition of these processes would likely prove detrimental for melioidosis treatment. Such inhibition could impair the restriction of bacterial replication at various level, as observed in the absence of MyD88 (Wiersinga et al., 2008c), caspase-11 (Broz et al., 2012), PAI (Kager et al., 2011), and A2AP (Kager et al., 2012), and predispose to hemorrhage. Approaches targeted more specifically to NLRP3, IL-1β, neutrophil elastase, bradykinin, TF and coagulation may result beneficial if achieved in a context where a protective physiologic inflammatory response and coagulation are not disrupted (see Table 2 and Figure 1).

**Table 2:** Potential therapeutic drugs for melioidosis treatment.

Drug	Target	Mechanism and effect	Models/clinical trial	References
Anakinra	IL-1 receptor	Inhibits IL-1 receptor signaling	Mouse pneumococcal pneumonia; clinical trials for several inflammatory diseases.	Dinarello, 2003
MCC950	NLRP3	Best characterized NLRP3 inhibitor. Binds to NACHT domain and stabilizes NLRP3 in inactive conformation. Selective, broad-spectrum NLRP3 inhibitor, blocks IL-1b maturation.	Preclinical studies in mouse; Phase II clinical trial for rheumatoid arthritis. Discontinued due to hepatotoxicity.	Ma et al., 2025; Mangan et al., 2018
Glyburide	NLRP3	Inhibition of PAMP-, DAMP- or crystal-dependent NLRP3 activation. Decreased diseased severity in melioidosis patients under Glyburide treatment.	Mouse studies. BMM stimulation and LPS shock model; Phase II clinical trial for traumatic brain injury.	Ma et al., 2025; Koh et al., 2011b; Kewcharoenwong et al., 2016; Koh et al., 2013; Lamkanfi et al., 2009
Sivelestat	Neutrophil elastase	Competitive NE inhibitor. improved survival, decreased bacterial burden, inflammation, lung injury and vascular damage.	Mouse studies; *Streptococcus pneumoniae* infection model and LPS-induced ALI; Clinical studies, severe bacterial pneumonia, ARDS.	Cui et al., 2020; Miyoshi et al., 2013
KRP-109		NE inhibitor. Improved survival and reduced inflammation	Mouse studies; *Streptococcus pneumoniae* infection model	
Pre-elafin		NE inhibitor. Decreased lung neutrophil influx and inflammation	LPS-induced ALI	
HOE-140	Bradykinin receptor B2	Bradykinin receptor B2 antagonist. Improved survival and decreased vascular permeability	Mouse melioidosis model; FDA-approved for acute hereditary angioedema	Sahoo et al., 2014; Nicola, 2017; Fein et al., 1997
DALBK	Bradykinin receptor B1	Bradykinin receptor B1 antagonist. Improved survival, decreased bacterial burden and neutrophil dysfunction	Mouse studies; CLP sepsis model.	Arifa et al., 2025
TFPI	Extrinsic coagulation pathway	Extrinsic coagulation pathway inhibitor. Improved survival and decreased coagulopathy.	Mouse and non-human primate sepsis models	Creasey et al., 1993; Carr et al., 1994; Opal et al., 2001
EDC34	Coagulation and antimicrobial	TFPI-2-derived peptide. Inhibition of contact system, enhancing of complement binding and direct microbicidal activity. Improved survival, decreased bacterial burden and lung injury.	Mouse studies; LPS-shock and Gram-negative infection.	Papareddy et al., 2013
H-D-Pro-Phe-Arg-CMK	Factor XII and plasma kallikrein	Contact system inhibition. Decreased lung injury and fibrin deposition.	Rat *Salmonella* infection model	Persson et al., 2000
Low molecular weight heparin	Coagulation	Anticoagulant. Improved survival, decreased lung injury and inflammation.	Mouse studies; CLP sepsis model; Clinical studies	Medeiros et al., 2023; Czempik and Wiorek, 2023

### 5.1 IL-1β inhibition

IL-1RA is a naturally occurring inhibitor of IL-1 and it has been used for many years for the treatment of arthritis and other autoimmune diseases. IL-1RA has also been employed for treatment of infections though results have been inconsistent. Given IL-1RA's safety profile and the harmful impact of IL-1β in melioidosis, it stands out as an excellent candidate for treatment of this disease. IL-1RA administration to *B. pseudomallei* infected mice was shown to decrease neutrophilic inflammation and increase survival (Ceballos-Olvera et al., 2011). A similar protective effect was observed using a blocking antibody against IL-1β (Weehuizen et al., 2016). IL-1RA administration was also found to be strongly protective against antibiotic resistant *E. coli* strains that cause acute pyelonephritis and bladder infection by reducing neutrophils activation and accelerating bacteria clearance (Ambite et al., 2023). Clinical studies indicate that treatment with IL-1RA can improve outcomes for patients with cystic fibrosis (Iannitti et al., 2016) and COVID-19 (Huet et al., 2020). In contrast, IL-1RA aggravated *S. aureus* induced septic arthritis (Ali et al., 2015). Importantly, prolonged IL-1RA use is not as strongly associated with increased susceptibility to infections as observed with other anti-cytokines regimens (Dinarello, 2003).

### 5.2 NLRP3 inhibition

A number of NLRP3 inflammasome inhibitors have been recently developed (see Table 2) and are being tested in various clinical trials, though toxicity may be an issue for some of these drugs. Numerous clinical reports have showed that NLRP3 activation is associated to exacerbated inflammation mediated by IL-1β and IL-18 in sepsis patients (Cui et al., 2020; Huang et al., 2022; Garnacho-Montero et al., 2020; Esquerdo et al., 2017) and suppression of NLRP3 activation during sepsis has received great attention (Ma et al., 2025). Interestingly, NLRP3 inflammasome inhibition in a peritoneal endotoxic mouse model was also found to decrease LPS-induced coagulation by inhibiting IL-1β and TF expression (Shi et al., 2022). Inhibition of NLRP3 activation in melioidosis may be beneficial by reducing production of IL-1β, which during *B. pseudomallei* infection is mostly dependent on NLRP3 (Ceballos-Olvera et al., 2011). This approach would still allow deployment of protective pyroptosis, which is predominantly triggered by NLRC4 in myeloid cells and by caspase-11 in neutrophils and epithelial cells (Wang et al., 2018; Kovacs et al., 2020; Oh et al., 2022). Because NLRP3 could also be activated downstream of caspase-11, its inhibition would further limit the caspase-11-dependent inflammation. One of the most convincing evidence that NLRP3 inhibition can be beneficial in melioidosis comes from studies of diabetic patients. Diabetes is identified as the most common risk factor for melioidosis. Glibenclamide (glyburide) is a broad-spectrum ATP-binding cassette transporter inhibitor and a K^+^ ATP-channel blocker (Nieland et al., 2004) often used to treat diabetes patients in Thailand. It was noticed that diabetic patients treated with this drug presented lower mortality rates and disease severity after contracting melioidosis (Koh et al., 2011a). This protective effect was associated to reduced pro-inflammatory cytokine production by neutrophils (Kewcharoenwong et al., 2016). A follow-up study using *B. pseudomallei* infection mouse model confirmed that glibenclamide protective role was associated with reduced neutrophil influx to the lung and decreased IL-1β secretion (Koh et al., 2013). The mechanism by which glibenclamide can decrease inflammation in diabetic patient was then clarified with the discovery that it inhibited NLRP3 inflammasome activation (Lamkanfi et al., 2009).

### 5.3 Neutrophil elastase inhibition

Supporting elastase inhibition as a viable therapeutic for melioidosis, the elastase inhibitor sivelestat decreased mortality of *B. thailandensis* infected mice (Sahoo et al., 2014). This effect could have been mediated by the decreased damage to the lung epithelial barrier as well as to the reduced vascular leakage due to elastase-mediated bradykinin release (Sahoo et al., 2014). Neutrophil elastase inhibition was shown to improve the host response to several other bacterial infections or in animal models of sepsis (Polverino et al., 2017; Domon and Terao, 2021). Neutrophil elastase inhibition with KRP-109 or sivelestat improved the survival and decreased lung inflammation and neutrophil infiltrate in severe pneumococcal pneumonia (Yanagihara et al., 2007; Yamada et al., 2011). Pre-elafin (another neutrophil elastase inhibitor) decreased neutrophil influx and inflammation in lung in LPS-induced acute lung injury model in mice (Vachon et al., 2002). Supporting the potential for clinical intervention in melioidosis, neutrophil elastase inhibitors increased survival of severe bacterial pneumonia and sepsis patients (Polverino et al., 2017; Nakamura et al., 2008; Miyoshi et al., 2013).

### 5.4 Bradykinin inhibition

Administration of HOE-140, a bradykinin antagonist, significantly increased survival in the melioidosis mouse model and decreased vascular permeability (Sahoo et al., 2014). Inhibition of bradykinin, or other contact system factors, can reverse severity of sepsis associated with bacterial infections (Nicola, 2017). Bradykinin inhibition improved the survival of patients with systemic inflammatory response syndrome and sepsis caused by Gram-negative infections (Fein et al., 1997). In line with these findings, it has been shown that deficiency or pharmacological inhibition (using DALBK) of Bradykinin receptor B1, but not B2, decreased neutrophil dysfunction and improved bacteria clearance and survival of mice in the cecal ligation and puncture (CLP) sepsis model (Arifa et al., 2025). Of note, BKRB2 is constitutively expressed in various tissues while BKRB1 is upregulated in response to inflammatory cytokines such as TNFα and IL-1β, highlighting its potential for therapeutic interventions in highly inflammatory conditions (Arifa et al., 2025; Rex et al., 2022; Shen and Zhang, 2023).

### 5.5 TF and factor XII inhibition

Although existing literature suggests that DIC could play a role in melioidosis, the specific contribution of TF and FXII in the disease's pathophysiology remains largely unexplored and further work in this area is needed before therapeutic strategies aimed at these pathways may be attempted. Targeting these pathways was proven beneficial in some bacterial infection models but not others.

#### 5.5.1 TF inhibition

Early studies using an *E. coli*-mediated septic shock model in baboons have reported that the use of anti-TF monoclonal antibody or treatment with TFPI protein improves survival and attenuate coagulopathy (Taylor et al., 1991; Creasey et al., 1993; Carr et al., 1994). Similar results were found for TFPI treatment in peritonitis sepsis model in other animal models (Goldfarb et al., 1998; Camerota et al., 1998) and in the CLP sepsis model in mice (Opal et al., 2001). A TFPI-2–derived peptide, EDC34, showed promise by reducing bacterial load and lung damage during severe systemic infections caused by *E. coli* and *P. aeruginosa* (Papareddy et al., 2013). In endotoxemia, anti-TF immunization was effective in preventing endotoxin lethal mortality (Dackiw et al., 1996). In contrast, treatment with rNAPc2, a selective inhibitor of the TF/FVIIa pathway, did not protect mice from *E. coli* peritonitis (Weijer et al., 2004).

#### 5.5.2 FXII inhibition

Studies have highlighted the potential benefits of inhibiting the activation of the contact system during bacterial infections. Pharmacological inhibition of FXII using a specific peptide inhibitor was shown to protect against lung injury in a rat model of pneumonia caused by *Salmonella* (Persson et al., 2000). Administration of a blocking antibody against FXII increased baboon survival in a model of disseminated intravascular coagulation (DIC) triggered by *E. coli* bacteremia (Pixley et al., 1993). These results underscore the potential therapeutic benefits of targeting the contact system in bacterial infections.

#### 5.5.3 Heparin treatment

Inhibition of coagulation through treatment with low molecular weight heparin (LMWH) decreased lung injury and inflammation and improved survival of mice and rats in the CLP sepsis model (Lu et al., 2013; Medeiros et al., 2023). In contrast, unfractionated heparin (UFH) treatment in *E. coli* pneumonic sepsis model did not protect mice and, at higher doses, promoted mortality (Li et al., 2011). In clinical studies, UFH decreased mortality of sepsis patients and LMWH treatment led to decreased mortality and thromboembolism in COVID-19 patients (Czempik and Wiorek, 2023). Other studies have also highlighted the benefit played by the immunomodulatory effect of heparin and not its anticoagulant capacity. Unfractionated heparin increased survival of mice in the CLP sepsis model by inhibiting apoE-mediated NKT activation and inflammation (Chuang et al., 2011) while anti-inflammatory partially-desulfated heparin (that has minimal anticoagulatory effects) improved survival, bacterial clearance and reduced lung injury in *P. aeruginosa* pneumonia in mice (Sharma et al., 2014).

## 6 Conclusion and perspective

Numerous studies have provided strong evidence supporting the targeting of the NLRP3 inflammasome and IL-1β in the treatment of melioidosis. It is important to recognize that significant differences have been reported between humans and mouse inflammasomes activation (Ariffin and Sweet, 2013; Wang et al., 2013) urging caution in translating basic discoveries to the clinic. For example, multiple NAIP molecules participate in NLRC4 activation in mice while only one exists in humans. It is also interesting that in human monocytes the NLRP3 inflammasome activation can follow an alternative pathway not present in mice. Several medications to inhibit IL-1β are already available and demonstrated excellent safety profiles in clinical use. Although their efficacy at mitigating sepsis has been so far disappointing, these failures may be due to a number of reasons including the different type of sepsis being treated, the patient diversity, and the time of administration emphasizing the importance for rapid diagnosis and precision medicine approaches (Cajander et al., 2024). The involvement of coagulation in the pathophysiology of melioidosis is not well understood, and further research in this area is needed before any strategies aimed at inhibiting this pathway can be safely implemented. One important aspect that needs to be clarified is the role played by caspase-11-dependent activation of TF in melioidosis. Does intravascular TF activation on monocytes/neutrophils affect the disease outcome differently than extravascular TF activation on endothelial cells/fibroblast? What is the contribution of the contact pathway to the disease process? How can we apply the knowledge gained from the endotoxic models, where the innate functions of coagulation may be irrelevant, to infections, where the inflammation and coagulation directly target the pathogen viability? A deeper understanding of these pathways could open new avenues for effective interventions, refining treatment options, and ultimately improving patient outcomes. Continued exploration in these areas will be crucial for advancing the therapeutic landscape for melioidosis.
